# Supplementary material for: GARP Regulates the Immune Capacity of a Human Autologous Platelet Concentrate
Source: Biomedicines. 2022 Dec 5;10(12):3136. doi: 10.3390/biomedicines10123136 (PMC9775012; doi:10.3390/biomedicines10123136)
Supplement: Supplementary file 1 [file biomedicines-10-03136-s001.zip › biomedicines-2026174-supplementary.pptx]

## Slide 1
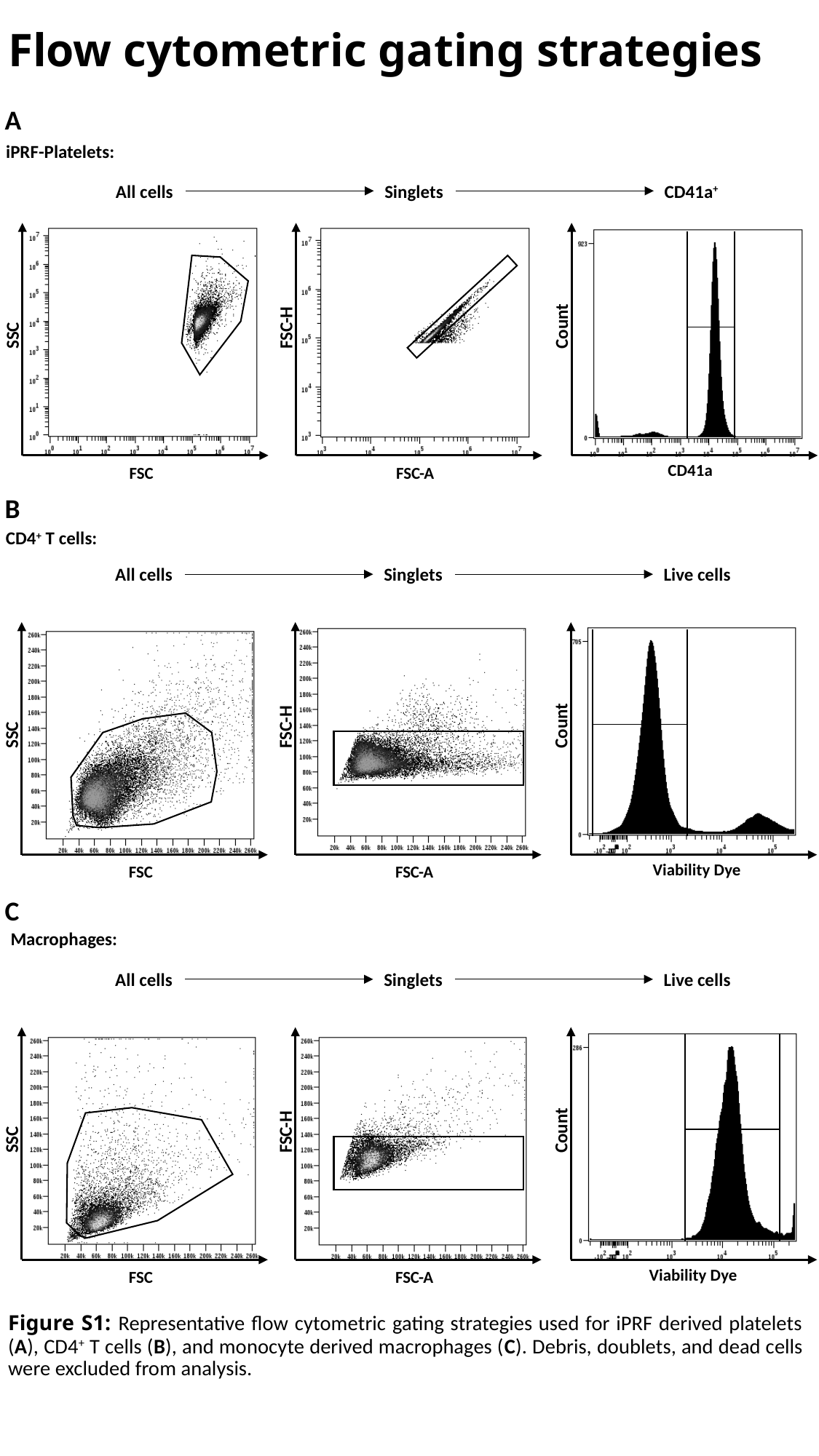

# Flow cytometric gating strategies
A
iPRF-Platelets:
CD41a+
All cells
Singlets
Count
FSC-H
SSC
CD41a
FSC
FSC-A
B
CD4+ T cells:
Live cells
All cells
Singlets
SSC
FSC
Count
FSC-H
Viability Dye
FSC-A
C
Macrophages:
Live cells
All cells
Singlets
Count
FSC-H
SSC
Viability Dye
FSC
FSC-A
Figure S1: Representative flow cytometric gating strategies used for iPRF derived platelets (A), CD4+ T cells (B), and monocyte derived macrophages (C). Debris, doublets, and dead cells were excluded from analysis.
